# The Clinical and Genetic Spectrum of 82 Patients With *RAG* Deficiency Including a c.256_257delAA Founder Variant in Slavic Countries

**DOI:** 10.3389/fimmu.2020.00900

**Published:** 2020-06-10

**Authors:** Svetlana O. Sharapova, Małgorzata Skomska-Pawliszak, Yulia A. Rodina, Beata Wolska-Kuśnierz, Nel Dabrowska-Leonik, Bozena Mikołuć, Olga E. Pashchenko, Srdjan Pasic, Tomáš Freiberger, Tomáš Milota, Renata Formánková, Anna Szaflarska, Maciej Siedlar, Tadej Avčin, Gašper Markelj, Peter Ciznar, Krzysztof Kalwak, Sylwia Kołtan, Teresa Jackowska, Katarzyna Drabko, Alenka Gagro, Małgorzata Pac, Elissaveta Naumova, Snezhina Kandilarova, Katarzyna Babol-Pokora, Dzmitry S. Varabyou, Barbara H. Barendregt, Elena V. Raykina, Tatiana V. Varlamova, Anna V. Pavlova, Hana Grombirikova, Maruša Debeljak, Irina V. Mersiyanova, Anastasiia V. Bondarenko, Liudmyla I. Chernyshova, Larysa V. Kostyuchenko, Marina N. Guseva, Jelena Rascon, Audrone Muleviciene, Egle Preiksaitiene, Christoph B. Geier, Alexander Leiss-Piller, Yasuhiro Yamazaki, Tomoki Kawai, Jolan E. Walter, Irina V. Kondratenko, Anna Šedivá, Mirjam van der Burg, Natalia B. Kuzmenko, Luigi D. Notarangelo, Ewa Bernatowska, Olga V. Aleinikova

**Affiliations:** ^1^Research Department, Belarusian Research Center for Pediatric Oncology, Hematology and Immunology, Minsk Region, Belarus; ^2^Department of Immunology, Children's Memorial Health Institute, Warsaw, Poland; ^3^Department of Immunology, Dmitry Rogachev National Medical Research Center of Pediatric Hematology, Oncology and Immunology, Moscow, Russia; ^4^Department of Pediatrics, Rheumatology, Immunology and Metabolic Bone Diseases, Medical University of Bialystok, Bialystok, Poland; ^5^Immunology Department, Pirogov Russian National Research Medical University, Moscow, Russia; ^6^Pediatric Immunology, Medical Faculty, Mother and Child Health Institute, University of Belgrade, Belgrade, Serbia; ^7^Centre for Cardiovascular Surgery and Transplantation, Brno, Czechia; ^8^Faculty of Medicine, Masaryk University, Brno, Czechia; ^9^Department of Immunology, University Hospital Motol, Prague, Czechia; ^10^Second Faculty of Medicine, Charles University, Prague, Czechia; ^11^Department of Pediatric Hematology and Oncology, University Hospital Motol, Prague, Czechia; ^12^Faculty of Medicine, Charles University, Prague, Czechia; ^13^Department of Clinical Immunology, Institute of Pediatrics, Jagiellonian University Medical College, Krakow, Poland; ^14^Department of Clinical Immunology, University Children's Hospital, Krakow, Poland; ^15^University Children's Hospital, University Medical Centre Ljubljana, Ljubljana, Slovenia; ^16^Faculty of Medicine, University of Ljubljana, Ljubljana, Slovenia; ^17^Pediatric Department, Faculty of Medicine, Comenius University, Bratislava, Slovakia; ^18^Department of Pediatric Hematology/Oncology and BMT, Wroclaw Medical University, Wroclaw, Poland; ^19^Department of Pediatrics, Hematology and Oncology Collegium Medicum in Bydgoszcz, Bydgoszcz, Poland; ^20^Nicolaus Copernicus University in Torun, Torun, Poland; ^21^Department of Pediatrics, Medical Center of Postgraduate Education, Warsaw, Poland; ^22^Department of Pediatric Hematology, Oncology and Transplantology, Medical University of Lublin, Lublin, Poland; ^23^Department of Pediatrics, School of Medicine, Zagreb Children's Hospital, University of Zagreb, Zagreb, Croatia; ^24^Faculty of Medicine, Josip Juraj Strossmayer University of Osijek, Osijek, Croatia; ^25^Department of Clinical Immunology, University Hospital Alexandrovska, Sofia, Bulgaria; ^26^Department of Pediatrics, Oncology and Hematology, Medical University of Lodz, Lodz, Poland; ^27^Department of Geographical Ecology, Belarusian State University, Minsk, Belarus; ^28^Department of Immunology, Erasmus MC, University Medical Center Rotterdam, Rotterdam, Netherlands; ^29^Laboratory of Molecular Biology, Dmitry Rogachev National Medical Research Center of Pediatric Hematology, Oncology and Immunology, Moscow, Russia; ^30^Department of Pediatric Infectious Diseases and Pediatric Immunology, Shupyk National Medical Academy for Postgraduate Education, Kiev, Ukraine; ^31^Pediatric Department, West-Ukrainian Specialized Children's Medical Center, Lviv, Ukraine; ^32^Consulting Center of Pediatric Medical Academy, St. Petersburg, Russia; ^33^Center for Pediatric Oncology and Hematology, Vilnius University, Vilnius, Lithuania; ^34^Hematology, Oncology and Transfusion Medicine Center, Vilnius University, Vilnius, Lithuania; ^35^Immunology Outpatient Clinic, Vienna, Austria; ^36^Laboratory of Clinical Immunology and Microbiology, Division of Intramural Research, National Institute of Allergy and Infectious Diseases, National Institutes of Health, Bethesda, MD, United States; ^37^University of South Florida at Johns Hopkins All Children's Hospital, Saint Petersburg, FL, United States; ^38^Massachusetts General Hospital for Children, Boston, MA, United States; ^39^Department of Clinical Immunology, Russian Clinical Children's Hospital by Pirogov Russian National Research Medical University, Moscow, Russia; ^40^Department of Immunology, Erasmus MC, University Medical Center Rotterdam, Rotterdam, Netherlands; ^41^Department of Pediatric, Laboratory for Pediatric Immunology, Willem Alexander Children's Hospital, LUMC, Leiden, Netherlands; ^42^Department of Epidemiology and Monitoring of Primary Immunodeficiencies, Dmitry Rogachev National Medical Research Center of Pediatric Hematology, Oncology and Immunology, Moscow, Russia

**Keywords:** *RAG1*, *RAG2*, primary immunodeficiency, geographic distribution, incidence, Slavic children

## Abstract

**Background:** Variants in recombination-activating genes (*RAG*) are common genetic causes of autosomal recessive forms of combined immunodeficiencies (CID) ranging from severe combined immunodeficiency (SCID), Omenn syndrome (OS), leaky SCID, and CID with granulomas and/or autoimmunity (CID-G/AI), and even milder presentation with antibody deficiency.

**Objective:** We aim to estimate the incidence, clinical presentation, genetic variability, and treatment outcome with geographic distribution of patients with the *RAG* defects in populations inhabiting South, West, and East Slavic countries.

**Methods:** Demographic, clinical, and laboratory data were collected from *RAG*-deficient patients of Slavic origin via chart review, retrospectively. Recombinase activity was determined *in vitro* by flow cytometry-based assay.

**Results:** Based on the clinical and immunologic phenotype, our cohort of 82 patients from 68 families represented a wide spectrum of *RAG* deficiencies, including SCID (*n* = 20), OS (*n* = 37), and LS/CID (*n* = 25) phenotypes. Sixty-seven (81.7%) patients carried *RAG1* and 15 patients (18.3%) carried *RAG2* biallelic variants. We estimate that the minimal annual incidence of *RAG* deficiency in Slavic countries varies between 1 in 180,000 and 1 in 300,000 live births, and it may vary secondary to health care disparities in these regions. In our cohort, 70% (*n* = 47) of patients with *RAG1* variants carried p.K86Vfs^*^33 (c.256_257delAA) allele, either in homozygous (*n* = 18, 27%) or in compound heterozygous (*n* = 29, 43%) form. The majority (77%) of patients with homozygous *RAG1* p.K86Vfs^*^33 variant originated from Vistula watershed area in Central and Eastern Poland, and compound heterozygote cases were distributed among all Slavic countries except Bulgaria. Clinical and immunological presentation of homozygous *RAG1* p.K86Vfs^*^33 cases was highly diverse (SCID, OS, and AS/CID) suggestive of strong influence of additional genetic and/or epigenetic factors in shaping the final phenotype.

**Conclusion:** We propose that *RAG1* p.K86Vfs^*^33 is a founder variant originating from the Vistula watershed region in Poland, which may explain a high proportion of homozygous cases from Central and Eastern Poland and the presence of the variant in all Slavs. Our studies in this cohort of *RAG1* founder variants confirm that clinical and immunological phenotypes only partially depend on the underlying genetic defect. As access to HSCT is improving among RAG-deficient patients in Eastern Europe, we anticipate improvements in survival.

## Introduction

Recombination-activating gene 1 (*RAG1*) and 2 (*RAG2)* encode lymphoid-specific proteins that are expressed during the early stages of T-cell and B-cell development and initiate the process of V(D)J recombination by introducing DNA double-strand breaks (DSBs) for recognizing millions of possible antigens (1). Genotype–phenotype correlation is strong, as null variants of *RAG1* and *RAG2* genes result in the T-B- severe combined immune deficiency (SCID) phenotype, whereas hypomorphic *RAG* variants have been associated with distinct clinical entities including Omenn syndrome (OS) and combined immunodeficiency with granuloma and/or autoimmunity (CID/G-AI) with herpesvirus infections and lymphoproliferation (2–5) and with selective polysaccharides antibody deficiency (6). Furthermore, in the era of widespread next-generation sequencing, RAG deficiency is being identified among adults with variants of antibody deficiencies with a frequency of 1 in 500 patients (7).

The *RAG1* and *RAG2* genes are polymorphic. Described clinical phenotypes are associated with a variety of variants including non-sense, frameshift, in-frame deletion or insertion, and missense variants of the *RAG1* and *RAG2* genes that affect various domains of the proteins (1). Among numerous *RAG* variants, some of them were observed in a Jewish population with a high rate of consanguineous marriages (8). In our previous report of 11 OS patients from the East Slavic regions, we described the high rate of c.256_257delAA (p.K86Vfs^*^33) in the *RAG1* gene (*n* = 4, 50%) (9). This variant was also observed in Polish and Serbian patients with OS and SCID phenotypes (10–13), which suggests a founder effect.

Currently, there is no published systematic evaluation of Slavic patients with *RAG* deficiency. In this report, we aim to describe the genetic landscape of *RAG* deficiency by estimating the incidence, genetic diversity, clinical and immunological presentation, and survival rate of a large cohort of Slavic patients. In addition, we focus on *RAG1* p.K86Vfs^*^33 as a candidate founder variant among Slavic patients by studying the geographic distribution of allele and genotype frequencies of *RAG1* p.K86Vfs^*^33 mutation among patients in major Slavic populations.

## Materials and Methods

### Patients and Kindreds

Patients with pathogenic *RAG* variants were recruited retrospectively for this study through extensive collaboration with clinical immunologists who collected the data of national primary immunodeficiency (PID) registries from *East Slavs* (Russia, Belarus, and Ukraine), *West Slavs* (Poland, Czech Republic, and Slovakia), and *South Slavs* (Serbia, Slovenia, Montenegro, Croatia, and Bulgaria). The patients were divided into ethnic groups (East, West, and South Slavs) according to their country of origin.

### Ethics Statement

Informed consent forms were signed by the parents as requested and approved by the institutional review boards of various institutions involved. The protocol of study was approved by the institutional review board of Belarusian Research Center for Pediatric Oncology, Hematology, and Immunology (IRB0012-2015).

### Study Design

A detailed questionnaire was completed by the physicians including demographic data (gender, country, place of birth, and year of birth), variants, and clinical data (age at disease manifestation, age at diagnosis, clinical and immunologic phenotype, and outcome).

### Assignment to Phenotypic Subgroups

Corresponding clinicians from each country assigned patients to one of the following subgroups—SCID, OS, atypical SCID (AS), or combined immunodeficiency (CID)—on the basis of clinical and immunologic phenotype, and age at manifestation.

The Primary Immunodeficiency Treatment Consortium (PIDTC) diagnostic subdivides SCID into three categories: typical SCID, atypical SCID, and OS, based on total T-cell count, lymphocyte proliferation, presence of maternal T cells, characteristic phenotypic features, and gene defects. CID was classified by late and mild clinical presentation according to published criteria (4, 14). Roifman et al. distinguished CID from SCID based on a total CD3+ T-cell count of >500/μl (15). The 2019 ESID criteria for diagnosis of CID requires a symptomatic patient (infections, immune dysregulation) or history of affected family members with immune phenotype of two of the four parameters (low CD3 or CD4 or CD8 T cells, low naïve CD4 and/or CD8 T cells, elevated γδ T cells, and reduced proliferation to mitogen or TCR stimulation). This ESID 2019 criterion does not discuss underlying genetic defects for CID patients (16). The IUIS 2020 classification does list genetic defects for CID but fails to include *RAG* deficiency in this category (17).

To estimate the number of newborns with *RAG* variant in each country, the information about affected siblings in families with *RAG* variants was collected through hospital or personal records of affected families.

### Mapping of Variants

Mapping was performed by the *ArcGIS 10.5* program on the map of Central–Eastern Europe and Russia.

### *RAG1/RAG2* Sequencing

Gene sequencing was performed using standard techniques (panel-based, Sanger) in local laboratories in Europe. The reference DNA and protein sequences for *RAG1* are from NIH RefSeq NM_000448.2 and NP_000439.1 and those for *RAG*2 are from NM_001243785.1 and NP_001230714.1, respectively.

### Measurement of Recombination Activity

For single-allele mutation, assay is based on a v-Abl *Rag1*/*Rag2*^−/−^ pro-B cell line containing a single pMX-INV integrated cassette (18).

### TRECs and KRECs

TRECs and KRECs quantity was measured by RQ-PCR using plasmid standards, and ALB was taken as an internal control according to manufacturer instructions.

### Statistical Analysis

Allele frequencies were compared by chi-square test or Fisher's exact tests. Kaplan–Meier curve was computed for survival post-hematopoietic stem cell transplant (HSCT). *p* < 0.05 was considered significant. Statistical analysis was performed using GraphPad Prism version 6.0 (GraphPad Software Inc., San Diego, CA, USA).

## Results

### Population Demographics

We retrospectively collected and studied 82 patients with RAG deficiency from 67 families in 12 countries, born in the period of 1992–2018. Thirty-five of 82 (43%) patients were reported previously (9–13, 19–23). Distribution was even between males (*n* = 40, 49%) and females (*n* = 42, 51%). The median age at diagnosis of *RAG* was 1.3 years (range, 1 day to 24 years).

At the time of manuscript submission (March 2020), 43 of the 82 patients died (52%, 8 out of 43 after HSCT). All patients alive received HSCT (ranging from 1 to 18 years) except three females with LS/CID presentation from Poland (42_f), Bulgaria (54_f), and Czech Republic (55_f) who are alive without transplantation at the ages of 3, 6, and 25 (Supplementary Tables E1 and E2 in this article's Online Repository).

The largest group of patients (*n* = 40, 48.8%) was West Slavs from Poland (*n* = 29, *n* = 1 from Lithuania, Polish origin), Czech Republic (*n* = 8) and Slovakia (*n* = 2). The second largest group was East Slavs (*n* = 30, 36.5%) from Russia (*n* = 24), Ukraine (*n* = 4), and Belarus (*n* = 2). The smallest group was South Slavs (*n* = 12, 14.6%) from Serbia (*n* = 6), Slovenia (*n* = 3), Croatia (*n* = 1), Montenegro (*n* = 1), and Bulgaria (*n* = 1).

### Clinical, Immunological, and Genetic Phenotypes

All patients have genetically confirmed pathogenic *RAG1 or RAG2* variants. *RAG1* variants were detected in 67 patients in 54 families (82%) and *RAG2* variants were detected in 15 patients from 13 families (18%). Nineteen children (23%) out of 16 families and 8 children (10%) out of 7 families were homozygotes while 47 patients (59%) out of 39 families and 8 patients (10%) out of 6 families were compound heterozygotes for *RAG1* and *RAG2* variants, respectively (Supplementary Tables E1 and E3).

**Figure 1:**
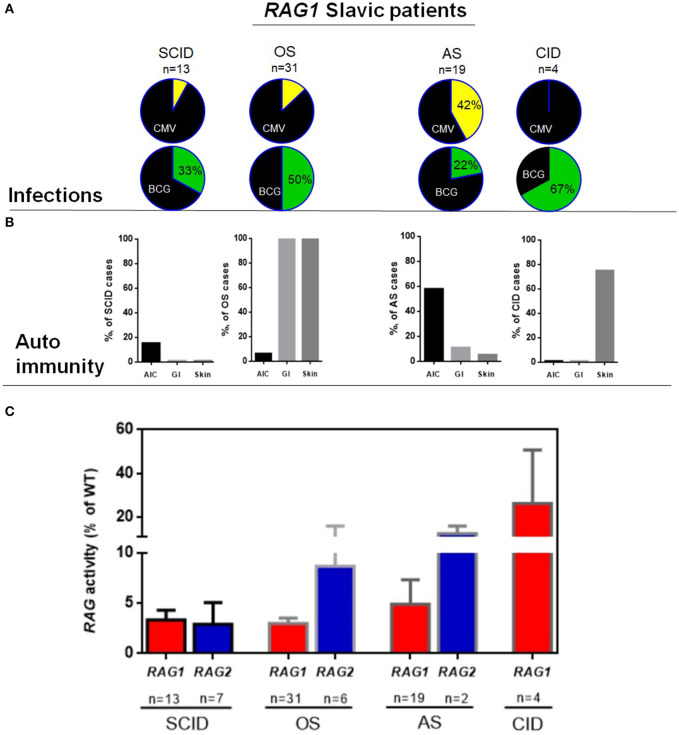
Characteristics of RAG Slavic cohort. **(A)** Infectious complications in *RAG1* patients, upper row CMV in four groups, lower row BCG complications among patients, who received BCG vaccination. **(B)** Frequency of autoimmunity in 4 groups of *RAG1* deficiency. **(C)** Recombinase activity of *RAG1/RAG2* variants for the 82 patients divided into four major clinical presentations for *RAG1* and three clinical presentations for *RAG2*.

Clinical phenotypes, as determined by attending clinician following immunological and clinical guidelines, included SCID (*n* = 20), OS (*n* = 37), AS (*n* = 21), and CID (*n* = 4) with highly mixed immune phenotypes (different numbers of T cells in all groups and domination of B+ phenotype in AS group); however, *in vitro* relative recombinase activity showed good correlation between genotype and clinical cathegories. (Figure 1C, and Supplementary Tables E1 and E3 in this article's Online Repository). Four (10%) out of 41 vaccinated SCID patients with RAG1/2 variants developed BCGosis infection, and 12 (3%) BCGitis were noted. As expected, the AS group had increase frequency of autoimmune cytopenias and history of herpesvirus infections (Figure 1A,B and Supplementary Table E2).

### Patients With RAG1 Variants

Patients with RAG1 variants are described in Supplementary Tables E1 and E2. Most patients presented with OS (46%) (31 children from 26 families) followed by AS (28%) (19 patients from 16 families), SCID (19%) (13 patients from 11 families), and CID (6%) (4 patients from 4 families).

Patients diagnosed as having OS and SCID were characterized by classical clinical and immunologic phenotype (Supplementary Table E1). Their clinical phenotype allowed for diagnosis before the age of 1 year. On the other hand, the median age at the time of diagnosis in AS and CID was 4 years (± 6.02 years) and ranged from 5 months to 24 years (Supplementary Tables E1 and E2).

In the AS group, infectious complications were notable for high prevalence of CMV infection (*n* = 9, 42%) in both localized (*n* = 2, retinitis) and systemic forms (*n* = 7). Among patients who received BCG vaccination, only a fraction (4 of 18, 22%) developed BCG infections (five with BCGitis and one with BCGosis) (Supplementary Table E2 and Figure 1A).

Autoimmune findings in the AS and CID group were observed in 12/19 and 4/4 of patients (Supplementary Table E2), with autoimmune cytopenia being most prevalent in AS (*n* = 11/19, 58%) including autoimmune hemolytic anemia (*n* = 8), isolated immune thrombocytopenia (ITP) (*n* = 2), and multiple lineage cytopenia (*n* = 2). Autoimmune colitis was diagnosed in 9% (2/19) of AS patients with autoimmunity. In the CID group, one patient had skin granuloma, one developed vasculitis, and one had vitiligo (Supplementary Table E2 and Figure 1B).

Immunological phenotype of the AS group is summarized in Supplementary Table E1. Curiously, high fraction of the patients had preserved fraction of B cells (>65% in 13 of 19 patients) at the time of diagnosis. However, 2 of 7 (28%) patients had complete absence of KRECs. This suggests abnormal B cell maturation even in the setting of preserved fraction of total B cells. Four patients presented with normal IgG level, and one had IgA deficiency at the age of 4 years that progressed to agammaglobulinemia with age.

In this study, we additionally determined the recombination activity of patient's RAG1/RAG2 mutant proteins. Of the *RAG* variants, 38 variants were unique, including frameshift (*n* = 7), non-sense (*n* = 4), and missense (*n* = 27), and 23 *RAG1* mutations were new in our cohort, and for all of them, recombination activity was determined (Supplementary Tables E1 and E2). We had the possibility to demonstrate that mutations associated with SCID and OS had lower residual activity than mutations detected in patients with less severe clinical presentations (AS and CID) (Figure 1C). Nevertheless, genotype–phenotype correlation was less evident in our cohort; we have not found significant differences between OS/SCID and AI/CID in clinical and laboratory markers. Among the patients with homozygous *RAG1* p.K86Vfs^*^33 (c.256_257delAA) variant (*n* = 18), the clinical and immunological presentation was highly diverse, 4 patients presented as OS, 1 as SCID, and 13 as AS (Supplementary Tables E1 and E2); these findings were supported by Supplementary Table E1 Pasic et al. (12). This is suggestive of the strong influence of other genetic and/or epigenetic factors in shaping the final clinical and immunological phenotype.

### *RAG1* Variants

Homozygous *RAG1* variants were detected in 18 families, but consanguinity was identified only in 4 (22%) [3 families from Poland (2a_m, 2b_m; 39_f; 41a_m, 41b_m) and 1 from Serbia (44a_m, 44b_f, 44c_m)].

**Figure 2:**
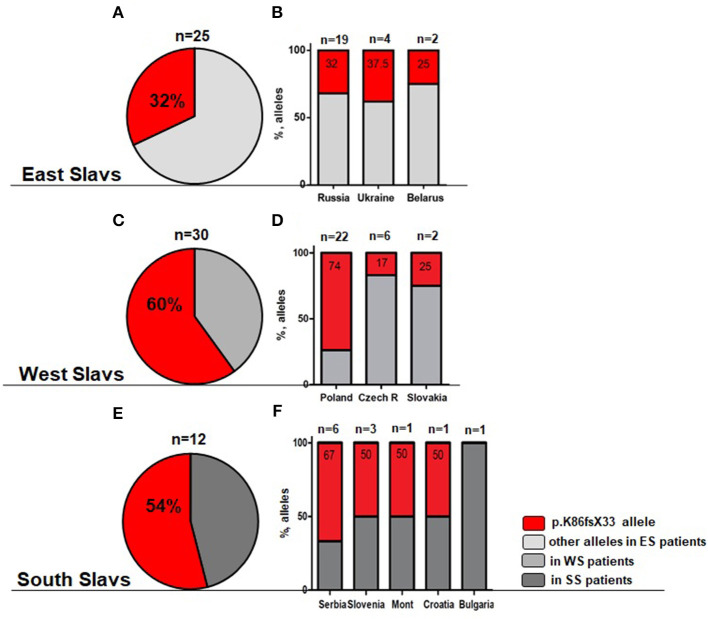
Overall frequency of c.256_257delAA allele (p.K86Vfs*33) among all cohort of *RAG1* patients. **(A,B)** Light gray—East Slavs (ES); **(C,D)** Gray—West Slavs (WS); **(E,F)** Dark gray—South Slavs (SS); **(B,D,F)** Percentage of p.K86Vfs*33 allele in studied countries. In Montenegro and Croatia, only one patient was studied.

The most frequent genetic *RAG1* anomaly among patients of all groups and in 10 out of 11 countries was *RAG1* p.K86Vfs^*^33 (c.256_257delAA) (Supplementary Table E1). This deletion occurred in one or both alleles in 22 out of 30 patients with OS, in 7 out of 13 SCID patients, and in 16 out of 19 patients with AI and 2/4 CID phenotype. Because of the enrichment of the p.K86Vfs^*^33 variant among all cohorts, we calculated the frequency of this allele among East, West, and South Slavic patients (Figures 2A–F) and their families (data not shown). Nine families had two or three children with immunodeficiency, and therefore, we conducted a separate analysis of families and analyzed frequency between countries counting one case per family.

Patients of South and West Slavic origin demonstrated the highest frequency of c.256_257delAA allele (Figures 2C,E). We analyzed the relative frequency of this predominant allele among Slavic patients and families. The differences in the allele frequency of c.256_257delAA frequency among the patients (χ^2^ = 8.4; *p* = 0.015) and the families (χ^2^ = 22.8; *p* < 0.0001) of the East/West/South Slavs proved to be statistically significant. In pairwise comparisons, the West Slavic population had a higher relative frequency of this allele than the East Slavic (families *p* = 0.0006; patients *p* = 0.006), but it did not differ from the South Slavic population (families *p* = 0.14; patients *p* = 0.81).

Besides the analysis of different Slavic cohorts, we calculated the contribution of each country to the overall frequency of *RAG1* c.256_257delAA allele and showed the highest occurrence in Poland (Figure 2D). *RAG1* c.256_257delAA allele (p.K86Vfs^*^33) was present in over 50% of *RAG1*-deficient patients from Serbia, Slovenia, Montenegro, and Croatia, but extrapolating this finding to the whole South Slavic population is premature because only one patient was from Croatia and Montenegro (Figure 2F).

Among East and South Slavs, we observed only two families (one from Ukraine and one from Serbia) where p.K86Vfs^*^33 was detected in a homozygous constitution, but consanguinity was proved only in the Serbian patients. The Polish cohort of families showed 12 homozygous genotypes among 18 *RAG1* positive families, with only three cases of proven consanguinity.

**Figure 3:**
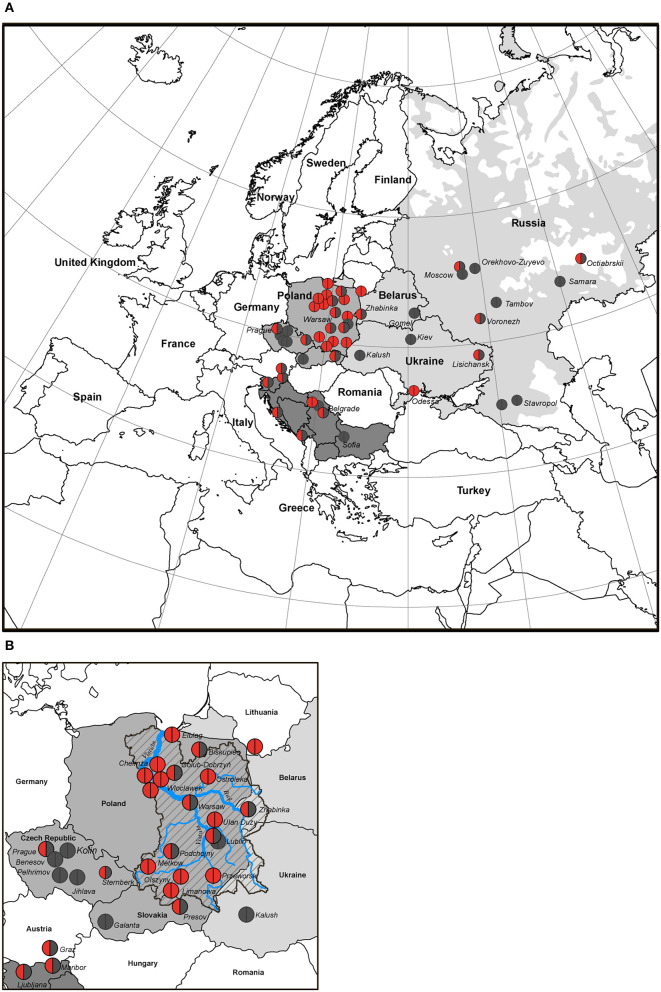
Distribution map of families with *RAG1* p.K86Vfs*33 variant in Slavic countries (50 of 55 families). **(A)** The birthplace of the patients is indicated by the location of the circles; homozygous p.K86Vfs*33 variant is represented by red circles; heterozygous p.K86Vfs*33 variant is half red/half gray, and other variants are gray. **(B)** Map of the Western Slavs; the Vistula River basin is overlaid on the map of Poland. The blue line is the Vistula River, a zone marked by an oblique black line—the Vistula River basin, the geographic area coincides with the region of the largest concentration of families where patients with p.K86Vfs*33 homozygous variants were born.

Due to the high frequency of the *RAG1* p.K86Vfs^*^33 variant in Poland and homozygous genotypes, we studied its geographical distribution in West, East, and South Slavic populations. Our findings are illustrated on the map of East Europe and Russian Federation based on the patient's place of origin (one patient from each family) (Figure 3).

**Figure 4:**
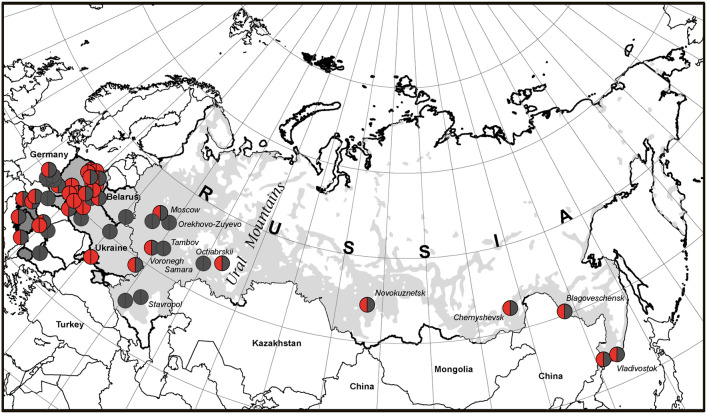
The geographic picture of the p.K86Vfs*33 distribution among East Slavs. Light gray zone on the map of Russia is the territory of predominant Russian settlement (24).

We studied the distribution of *RAG1* p.K86Vfs^*^33 variant in 11 out of 13 Slavic ethnic groups and it was widespread and found in 10 out of 11 countries except Bulgaria (Figure 3). The occurrence of p.K86Vfs^*^33 was observed from Ljubljana (Slovenia) in the West to Vladivostok in the East and from Moscow in the North to Cetinje (Montenegro) in the South (Figures 3, 4). One patient who originated from Slovenia was born in Austria (Graz), but after delivery, he was treated in the homeland of Slovenia (Figure 3).

The geographic center of the presumed origin of the *RAG1* p.K86Vfs^*^33 variant corresponds to the Vistula watershed area of Central and Eastern region in Poland in which the variant was the most frequent (Figure 3B) and suggestive of shared origin for the p.K86Vfs^*^33 alleles in all Slavic populations.

Due to a small number of cases, gradual decrease in the prevalence of p.K86Vfs^*^33 among East and South Slavs could not be determined, but it is clearly shown that heterozygous families are spread among other Slavic countries from the Polish area of homozygosity (Figure 3).

The broadest area of East Slavs in Russia is located mainly in the European part of the Russian Federation (25). In our cohort of patients from Russia, the majority (11/19) is concentrated on the territory west of the Ural Mountains, but 8 patients from 5 families were born in Siberia and the Russian Far East, and all inherited our studied variant (Figure 4). This finding does not contradict our assumption that p.K86Vfs^*^33 has a Slavic origin. According to the map of the resettlement of peoples in Russia, most Russians live in the central part or in the south and northwest of Russia as well as in the Ural region. To the east of the Ural Mountains, Russians live predominantly near the southern border of Russia, which is shown in Figure 3 and in Figure 4 as a light gray area on the white map of the Russian Federation.

### Patients with RAG2 Variants

Patients with RAG2 variants were detected only in three countries among West (Poland and Czech Republic) and East Slavs (Russia) (Supplementary Table E3). Among 15 patients from 13 families, 7/15 presented as SCID, 6/15 presented as OS, and 2/15 presented as AS. Two Polish females manifested as atypical SCID/CID and one had a sister with SCID (8a_ f). In one Russian family, one male presented as OS (3b_ m) and one presented as SCID (3a_ m).

Females with late manifestation of *RAG2* variant presented with encephalitis, HSV, bronchitis, pneumonia, neutropenia and pneumonia (8b_f), local BCGitis, and failure to thrive (12_f).

Interestingly, the obtained data showed that 53% (8/15) of patients had homozygous variants in the *RAG2* gene, but consanguinity was established only in one family history (1_m). Despite the marked diversity of the genotypes, there are some repeated variants, p.Y434H in Russian, p.R229Q in Polish and Czech children (in homo and in heterozygous state), and p.W453R only among Polish patients. However, an obvious dominating region among variants was not established due to a small number of the studied cases.

The *RAG2* variants in Slavic cohort showed a wide range of recombination activity (Supplementary Table E3). Mutations associated with severe combined immune deficiency and OS had lower activity than those detected in patients with atypical SCID presentations (Supplementary Table E3 and Figure 1D). Our data support genotype–phenotype correlation in *RAG2* deficiency in Slavic children (18).

### The Average Annual Incidence of Slavic Patients With *RAG* Deficiency and Survival

We examined the incidence of all types of *RAG* deficiency together (*RAG1/RAG2* genotype; OS/SCID/AS/CID phenotype) in each country during different periods. The average incidence of *RAG* deficiency was calculated based on the number of all diagnosed patients and their confirmed siblings and the number of live births in studied countries during the period from the year of the first registered patient in the country to the last one (Table 1). The average 10-year incidence was estimated by the number of all patients of Slavic origin diagnosed since 2008 to 2017 in each country (Table 1, last column).

**Table 1:** Average annual incidence of *RAG* deficiency in Slavic countries.

**No**	**Country**	**Number of patients with RAG**	**Number of families**	**Whole period**	**Sum of newborns for the period**	**The average annual incidence frequency**	Number of patients in 10 year period*	**Sum of newborns for the 10-years period**	The average 10-years incidence*
**EAST SLAVS**									
1	Russia	24	18	2002–2017	26 557 598	1:1 106 566	18	17 553 840	1:975 213
2	Ukraine	4	4	2004–2013	4 834 300	1:1 208 575	3	4 795 200	1:1 598 400
3	Belarus	2	2	1998–2009	1 145 223	1:572 611	1	1 127 193	-
**WEST SLAVS**									
4	Poland	28	23	1999–2017	7 201 693	1:257 203	11	3 904 235	1:354 930
5	Czech Republic	8	8	1993–2016	2 395 156	1:299 395	6	1 126 990	**1:187 832**
6	Slovakia	2	2	2007–2015	515 219	1:257 609	2	572 776	1:286 388
**SOUTH SLAVS**									
7	Serbia	6	3	1992–2011	1 529 226	1:254 871	1	671 049	-
8	Slovenia	3	3	2003–2013	223 206	**1:74 402**	1	212 986	-
9	Montenegro	1	1	1995	9 492	-	0	76 785	-
10	Croatia	1	1	2010	43 361	-	1	411 277	-
11	Bulgaria	1	1	2013	66 578	-	1	715 571	-

*Bold values indicate the highest average frequency of RAG deficiency*.

* *10-year period is 2008–2017*.

The obtained data showed that frequency of diagnosed *RAG* deficiency ranged from 1 per 75,000 in Slovenia to one per 1,100,000–1,600,000 live births in Russia and Ukraine.

**Figure 5:**
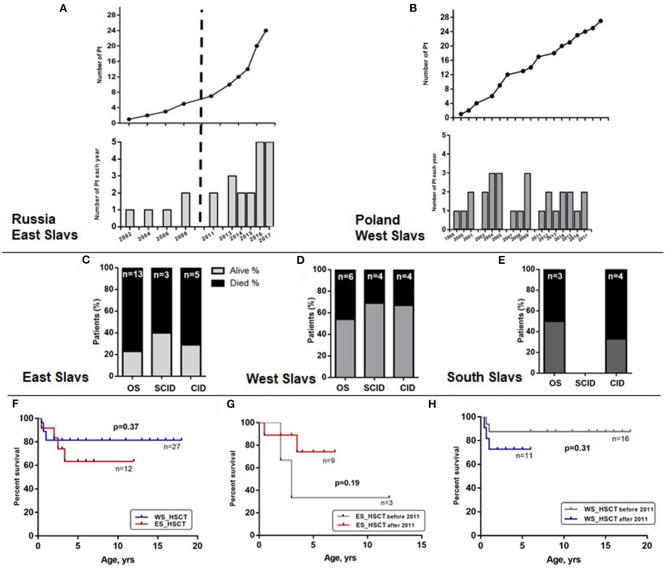
Identification and survival of Slavic patients. Number of patients diagnosed in Russia **(A)** and Poland **(B)**. Percentage of dead patients (black region) among clinical groups of patients with *RAG* variants and East **(C)**, West **(D)**, or South Slavic **(E)** origin. Overall survival displayed as Kaplan–Meier survival curve of WS and ES patient groups with HSCT **(F)**. Overall survival curve of ES **(G)** and WS **(H)** patient transplanted before and after 2011.

A wide range in frequency among Slavic countries suggest underdiagnosis of atypical late-onset cases. We compared the two largest *RAG* cohorts from Russia (*n* = 24) and Poland (*n* = 30). In further analysis, we graphically illustrated the number of diagnosed patients in the two countries (the age at diagnosis was added to the year of birth) and showed that Russia achieved great success in identification of *RAG* patients in the last 8 years (Figure 5A, lower). Exponential growth (Figure 5A, upper) was observed; 19 out of 24 *RAG* patients were diagnosed from 2011 to 2017, and their geography covered the entire country (Figure 4). Poland, as the largest West Slav country, uses advanced immunologic diagnostics services and one to three patients have been confirmed approximately each year since 1999 (Figure 5B).

Despite an increased awareness for SCID and OS variants and improved access for HSCT in most Slavic countries, the mortality rate of *RAG* patients is still high in Eastern Europe. We studied relative mortality rate among OS/SCID/CID groups of patients with *RAG* deficiency (Figure 5C—ES, Figure 5D—WS, and Figure 5E—SS). Neither comparison of transplanted patients in two groups (WS and ES) as biggest cohorts revealed any statistically significant difference in survival (Figure 5F). Additionally, we compared the results of HSCT before and after 2011, due to changing the protocols in last years and we were able to show that survival of East Slavic patients became much better after 2011 and reached the same percentage as in West Slavs (Figures 5G,H). We suggest that poor outcome in HSCT before 2011 (three out of five died after HSCT) in East Slavs was due to delayed diagnosis in AS/CID patients and associated complications (chronic infections and immune dysregulation) (Supplementary Figure E1C). Patients with OS were transplanted with a high rate of survival in ES/WS/SS (Supplementary Figure E1B) and with SCID in ES/WS countries (Supplementary Figure E1D).

## Discussion

Several cohort studies with international collaborators are published, and a recent review of literature enumerate the number of published cases with *RAG* deficiency over 400 (2, 4, 7–9, 13, 14, 23). Among the population with high rate of consanguity, founder variants for *RAG* genes were proposed among the Amish and in the Middle East (26–28).

In this study, we present the largest geographically defined cohort of patients with *RAG* deficiency. Our patients are predominantly of Slavic origin from 11 European countries retrospectively collected in a 27-year period (1992–2018). In our current and previous study of patients with *RAG1* p.K86Vfs^*^33 (c.256_257delAA), we confirmed that different clinical phenotypes can been found in patients with identical variants even within the same family (9, 13). Family studies for homozygous *RAG1* p.S480G and *RAG2* p.M459L were also reported in consanguineous marriages of Arabic descent (29, 30). Furthermore, similar non-familiar findings were reported with patients with *RAG1* c.519delT (p.E174SfsX27) (13). Among all these reports, our study with 18 homozygous patients *RAG1* p.K86Vfs^*^33 (c.256_257delAA) is the largest in the literature within a geographically linked population.

The data from California's SCID newborn screening identified *RAG1/2* variants in 28.6% of SCID/OS cases, with an incidence of about 1:250,000 (31). In populations with domination of consanguineous marriages, *RAG* deficiency was diagnosed in 48% (21/44) of Turkish (32) and 51% (19/37) of Iranian SCID patients (33). The incidence of *RAG* deficiency is difficult to establish, due to the highly variable phenotype, late age of manifestation of hypomorphic variants, and different level of diagnostics capacities in different countries (5). Recently, it was identified that RAG deficiency among adults with a variety of antibody abnormalities occurs with a frequency of 1 in 500 patients with any variant of antibody deficiency (7). That is much higher than the expected number of *RAG1/2* SCID or OS cases. Based on our data reported in this study, we determined that the minimal incidence of *RAG* deficiency varied from 1:189,000 in the Czech Republic to 1:1,200,000 in Ukraine; the average was 1 per 190,000–300,000 live newborns.

It is still unclear if all *RAG* patients with AS/CID phenotype (with residual recombination activity) can be detected by newborn screening (NBS) (14). Retrospective testing of NBS card showed absence of TRECs in case reports (34). Current observation showed that KREC level can support and underlying *RAG* deficiency is relevant and further justifies the consideration of KREC in universal NBS for SCID and AS/CID. Our retrospective data suggest that most of the patients with late-onset forms of *RAG* deficiency (CID) are likely missed, but the level of discovery of SCID/OS patients is reasonable in most Slavic countries. There is a great need for early identification of these patients before infectious and autoimmune complications occur. The availability of next-generation sequencing and the access to HSCT service make it possible to improve the outcome for this group.

In the present study, 27% of patients were classified by attending physicians as atypical SCID. This phenotype was first described by Schuetz C. et al. in 2008, which they named “atypical/leaky SCID.” During the last 10 years, there is growing awareness of these patients (2, 4, 7, 9, 13, 19, 20, 22, 30). Distinct clinical features include inflammatory and/or autoimmune complications. Autoimmune cytopenias (AIC) were the most frequent in our cohort, found in 58% (11/19 *RAG1* patients with AS phenotype). In the general population, ITP is the most common AIC. This is comparable to published data (23, 35). Regarding immunological features, although one third of the RAG1 patients had absence of very low B cell count, over two thirds of the cases had presence of low to normal B cells and immunoglobulins that progressive decline with age. Of note, many of our patients did not have full immune evaluation.

Slavs are the largest ethno-linguistic group in Europe (25, 36). In 10 out of 11 countries, the majority of patients carried p.K86Vfs^*^33 in RAG1 in homo or heterozygous state. Based on our data reported in this study, we established the geographic center of the origin of this specific RAG1 allele; it corresponds to the Vistula watershed area in Central and Eastern Poland (Figure 3B). Since SCID (Bernatowska E. unpublished data) and *RAG2* patients are evenly diagnosed in Poland, the lower frequency of absence *RAG1* variant p.K86Vfs^*^33 in the Western part of Poland may be likely related to distance from the geographic center of the founder variant or due to lower medical care in this region. Together, these data suggest that it is most likely that the founder *RAG1* variant p.K86Vfs^*^33 is of a Slavic origin.

Location of the Slavic homeland prior to their great expansion in the fifth to sixth centuries is one of the key questions of European history. Although it is assumed that prehistorically the original habitat of Slavs was Asia, from which they migrated in the third or second millennium BC to populate parts of Eastern Europe, a debate concerning the European homeland of Slavs seems to remain unsolved. Different theories concerning the Slavs' geographic origin based on archaeological, anthropological, and/or linguistic data have been formulated. One places the cradle of Slavs in the watershed of the Vistula and Oder rivers (present-day Poland), and the other locates it in the watershed of the middle Dnieper (present-day Ukraine) (32). The obtained data strengthen the hypothesis that the Slavs could originate from the watershed of the Vistula due to the high number of families with homozygous variant from that region (Figure 3B). However, at present, it is impossible to deny that the variant could be found in the watershed of the middle Dnieper due to low number of cases and low incidence of *RAG* diagnostics in the Ukraine. Additionally, the only homozygous variant among East Slavs was found in the Ukraine (Odessa), where the parents denied their consanguinity. Collectively, these data indicate that p.K86Vfs^*^33 could be established as a “Slavic” *RAG1* variant or a founder variant.

Population migrations in Europe have led to the distribution of ethnic groups and cultures, and consequently to genetic mixing. Migrations, together with other factors, have also determined the prevalence of genetic variants among other populations (25, 36). In the GnomAD database, this deletion was found in heterozygous state in six people of European population (non-Finnish) of 282,486 individuals [https://gnomad.broadinstitute.org/variant/11-36595109-TAA-T]. Reviewing published cases, we established that patients with homo- and heterozygous p.K86Vfs^*^33 *RAG1* variants were detected in European cohorts (2, 13, 37–40). A recent Turkey study reported one patient with homozygous p.K86Vfs^*^33 in *RAG1* among the cohort of 44 SCID patients (32) and a SCID infant with EBV-positive B-cell lymphoma of the liver with homozygous p.K86Vfs^*^33 was diagnosed in Austria (40). It should be noted that in all these previous studies, the ethnic origin of the patients was not documented.

Our study highlight that a founder RAG1 variant is frequently present in Eastern Europe, especially in the watershed area of the Vistula. As cases accumulate in a specific region in Poland, screening of carriers of pathogenic RAG variants may be of high importance and could be offered in case of parental interest to reduce the incidence of RAG deficiency in this population. Our study underscores the importance of region of birth and ethnic background in genetic diagnosis. Further studies are needed to explore the carrier frequency of p.K86Vfs^*^33 in Central and Eastern Poland, as well in otherSlavic countries.

## Data Availability Statement

The raw data supporting the conclusions of this article will be made available by the authors, without undue reservation, to any qualified researcher.

## Ethics Statement

Informed consent forms were signed by the parents as requested and approved by the institutional review boards of various institutions involved. The protocol of study was approved by the institutional review board of Belarusian Research Center for Pediatric Oncology, Hematology and Immunology (IRB0012-2015). Written, informed consent was obtained from the individual(s) for the publication of any potentially identifiable images or data included in this article.

## Author Contributions

SS designed the research, collected data, interpreted, analyzed the results and wrote the manuscript. DV prepared the maps. YY and TK studied RAG activity. MS-P, YR, BW-K, ND-L, BM, OP, PS, TF, TM, RF, AS, MS, TA, GM, PC, KK, SKo, TJ, KD, AG, MP, EN, SKa, KB-P, BB, ER, TV, AP, HG, MD, IM, AB, LC, LK, MG, JR, AM, EP, CG, and AL-P provided patient's information. JW, AS, TF, LN, OA, NK, and MB guided the writing of the manuscript. All authors have read and approved the contents of the manuscript.

## Conflict of Interest

MS-P, BW-K, ND-L, MB, AS, MS, KK, SK, TJ, KD, MP, KB-P, EB have served as a consultants, and/or speakers, and/or principal investigator/investigators, and/or have provided expert testimony for Baxter/Baxalta/Shire, CSL Behring, Grifols, Biotest, Octapharma and have received one or more payments for above activities. TA served as a speaker for Octapharma and Shire/Takeda. The remaining authors declare that the research was conducted in the absence of any commercial or financial relationships that could be construed as a potential conflict of interest. The reviewer YL declared a past co-authorship with one of the authors to the handling editor.
