# Supplementary material for: The Clinical and Genetic Spectrum of 82 Patients With RAG Deficiency Including a c.256_257delAA Founder Variant in Slavic Countries
Source: Front Immunol. 2020 Jun 10;11:900. doi: 10.3389/fimmu.2020.00900 (PMC7325958; doi:10.3389/fimmu.2020.00900)
Supplement: Supplementary file 4 [file Data_Sheet_1.docx]

**Supplemental data**

Figure S1

| 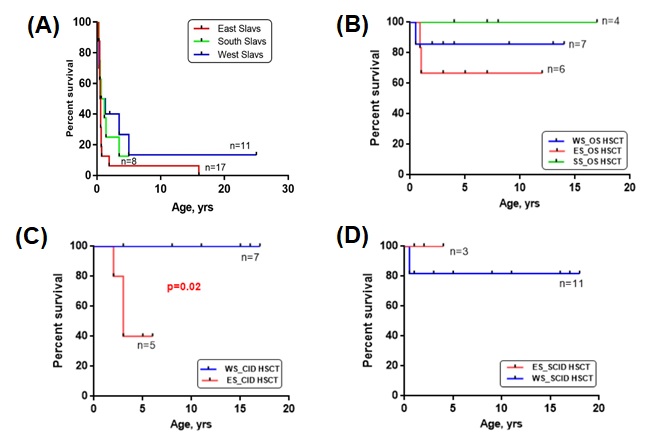 |
| --- |
| **Figure S1.** Overall survival displayed as Kaplan–Meier survival curve of WS, ES and SS patient groups without HSCT **(A)** and comparison of survival WS, ES, SS patients with OS **(B)**, with CID (C), with SCID **(D)** after HSCT |
